# Binding of [Au(dien)Cl]Cl_2_ to Tripeptides

**DOI:** 10.1155/MBD.1994.509

**Published:** 1994

**Authors:** Sabine L. Best, Milos I. Djuran, Peter Sadler

**Affiliations:** 1 Department of Chemistry Birkbeck College University of London 29 Gordon Square London WC1 OPP United Kingdom


					BINDING OF [Au(dien)Cl]Cl2 TO TRIPEPTIDES
Sabine L. Best, Milos I. Djuran and Peter Sadler
Department of Chemistry, Birkbeck College, University of London,
29 Gordon Square, London WC10PP, UK
Gold(Ill) is isoelectronic (5d8) and often isostructuml (square-planar) with Pt(ll) and might form the
basis of active anticancer agents [1]. However little is known about the biochemistry of Au(lll), a
subject also of interest in connection with the side-effects of antiarthritic gold drugs [2]. In this work
we have explored the binding of [Au(lll)(diethylenetriamine)CI]CI2 to tripeptides over the pH range
2-10.
Au(lll) forms strong complexes with these peptides, free and bound peptide exhibiting slow
exchange on the 1H NMR timescale. Binding to the imidazole ring of glycylglycyI-L-histidine (GGH)
occurs at pH's as low as 3, and in the pH range 3-6.5, three distinct species have been
characterized. Binding to the NH2 terminus of triglycine (GGG) is strong at pH >5. The preference
of Au(lll) for binding to amino groups, deprotonated amide nitrogen, carboxylate and amide oxygen
and histidine N and N of peptides will be discussed.
[1] P.J. Sadler, M. Nasr and V.L. Narayanan, in: Platinum Coordination Complexes in Cancer
Chemotherapy, Eds." M.P.Hacker, E.B. Douple and I.H. Krakhoff, Martinus Nijhoff,
Boston 1984, 290-304
[2] D. Schuhmann, M. Kubicka-Muranyi, J. Mirtschewa, J. G0nther, P. Kind, and E.
Gleichmann, J. Immunol. 145:2132-2139 (1990)
We thank the Wellcome Trust, Association for International Cancer Research, MRC, SERC, Royal
Society and ULIRS for their support.
509

				

